# Supplementary material for: Hierarchical small molecule inhibition of MYST acetyltransferases
Source: Nat Commun. 2026 May 13;17:4329. doi: 10.1038/s41467-026-70574-1 (PMC13172425; doi:10.1038/s41467-026-70574-1)
Supplement: Supplementary file 1 — Supplementary Information [file 41467_2026_70574_MOESM1_ESM.pdf]

Supporting Information

Hierarchical small molecule inhibition of MYST acetyltransferases

Xuemin Chen[1], Alexandra Castroverde[1], Minervo Perez[1], Ronald Holewinski[2], Kiall Suazo[1,2], Rashmi Karki[3], Thorkell Andresson[2], Benjamin Garcia[3], Jordan L. Meier[1]*

1. Chemical Biology Laboratory, National Cancer Institute, Frederick, Maryland, 21702, United States

2. Protein Characterization Laboratory, Frederick National Laboratory for Cancer Research, Leidos Biomedical Research, Frederick, Maryland, 21701, United States

3. Department of Biochemistry and Molecular Biophysics, Washington University School of Medicine, St. Louis, Missouri, 63110, United States

*To whom correspondence should be addressed: jordan.meier@nih.gov

Table of Contents for Supporting Information

	**Page**
Table of Contents	S2
Supplementary Figures	S2-S7
Uncropped scans of blots in supplementary figures	S8

Supplementary Figures

A

Incubation and 1st wash buffer	Aggregation reduction	Adhesion to the tube
Simple (PBS + protease inhibitor)	aggregate	in wash time
Simple + 0.1% NP-40	aggregate	in 4th wash time
Simple + 0.1% NP-40 +remove cell debris	reduced	no
Simple + 0.1% NP-40 +200 mM NaCl	reduced	no
Simple + 0.5% NP-40	aggregate	no
Simple + 1% NP-40+5% glycerol	aggregate	no

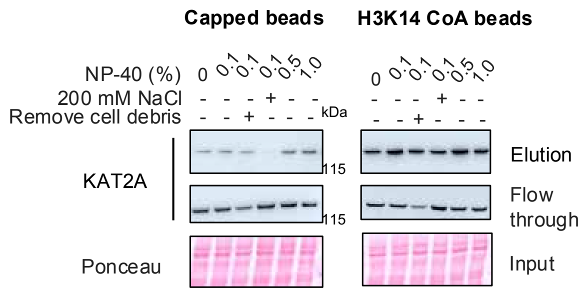

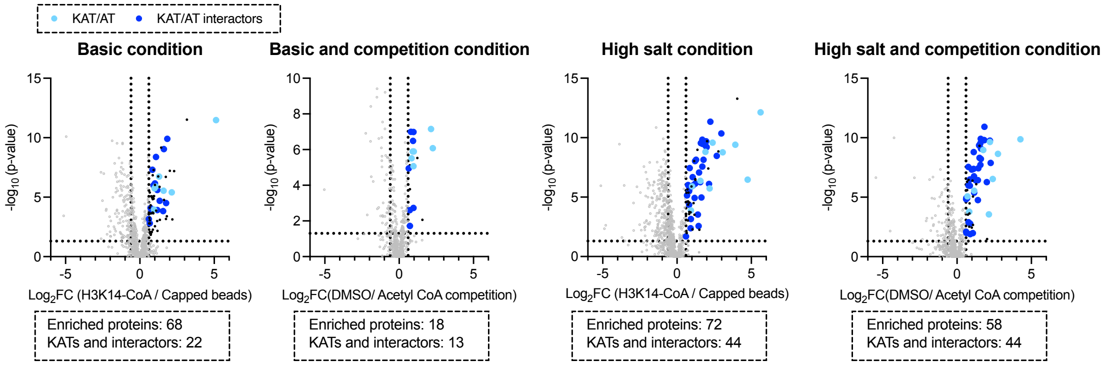

Figure S1. Optimization of the chemoproteomic affinity capture assay. (A) Experimental matrix used toe evaluate the effect of incubation buffer and first wash buffer composition on H3K14-CoA magnetic bead pull down assay efficacy. (B) Evaluating the effect of wash buffer on non-specific binding (capped beads) and KAT2A capture (H3K14-CoA beads). For each experiment, HeLa nuclear extracts (200 µL, 1 mg/mL) were centrifuged (20000 g, 30 mins, 4°C) to remove insoluble debris, followed by addition of 10 µL bead slurry and rotation (1 h, 4 °C). Beads were then washed with 500 µL of the indicated buffer and three times with a standard wash buffer (50 mM HEPES pH7.5, 150 mM NaCl, 1 mL). experiment was performed by 2 biological replicates. Elution indicates the captured protein eluted from beads, flow through indicates the protein present in the supernatant after incubation with beads. (C) LC-MS/MS proteomic analysis of effect of buffer conditions on KAT capture (*n* = 3 biological replicates). Basic condition: PBS, 1x protease inhibitor, 0.1% NP40 (incubation buffer, first wash buffer). High salt condition: PBS, 1x protease inhibitor, 0.1% NP40, 200 mM NaCl (incubation buffer, first wash buffer). Competition conditions refer to the same experiment performed on HeLa nuclear extracts that had been pre-incubated with acetyl-CoA (100 µM, 4 °C, 1 h). Cutoffs of $\log_2$fold-change >0.6 and p-value<0.05 were used to classify proteins as enriched.

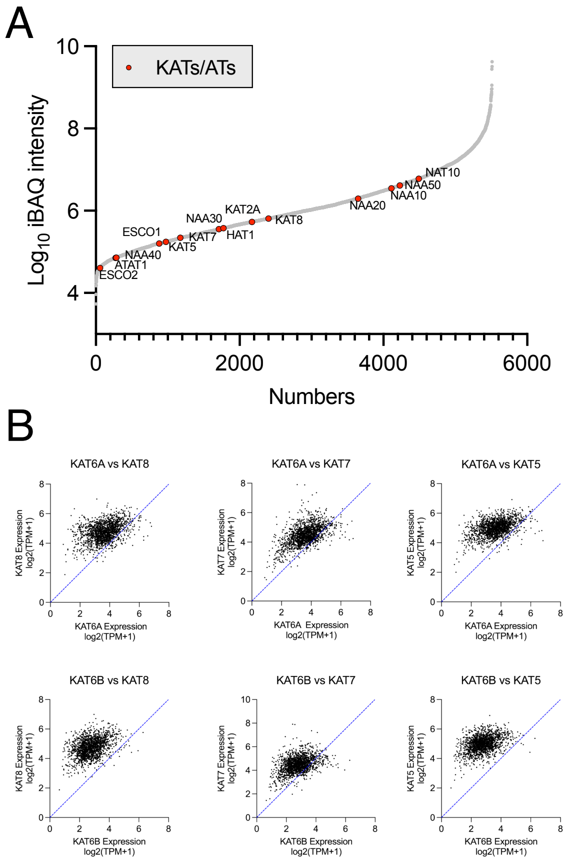

Figure S2. Expression comparison of KAT6A/B with other proteins. (A) MaxQuant analysis of protein abundance in Hela nuclear extracts by intensity-based absolute quantitation (iBAQ). HATs/ATs in HeLa nuclear extracts are shown in red (n=3 replicates). (B) Analysis of the relative KAT6A (top) and KAT6B (bottom) expression compared to other MYST family members (KAT8, KAT7, KAT5) across the DepMap cell line panel.

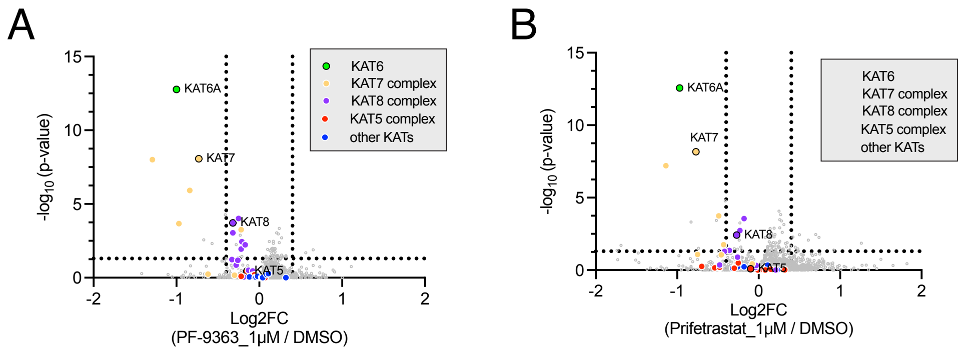

Figure S3. Chemoproteomic analysis of HeLa nuclear extracts with exogenous KAT6A spiked in under MYST inhibitor competition. (A) Proteome-wide competition analysis of PF-9363 competition in HeLa nuclear extracts with exogenous KAT6A spiked in (n = 3 biological replicates). Nuclear extracts were pre-incubated at the specified concentration (2 h, 4 °C) prior to KAT affinity capture. Vehicle DMSO pre-incubation was used as negative control. MYST KATs and interactors are color-coded according to complex. (B) Identical proteome-wide competition analysis performed with PF-8144 (aka prifetrastat,), n = 3 biological replicates.

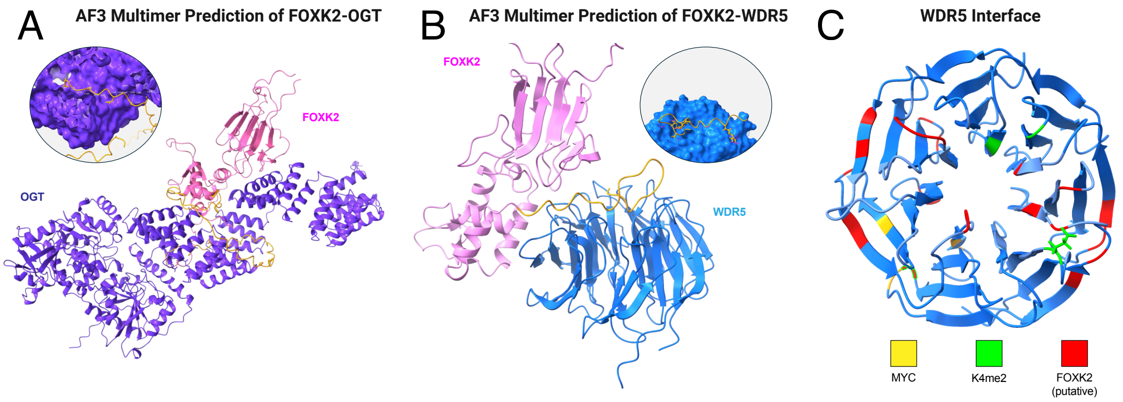

Figure S4. Predicted binding structure of FOXK2 with OGT or WDR5. (A) AF3-Multimer predicted structure of FOXK2 (pink) bound to OGT (purple). FOXK2 region predicted to interact with OGT is shown in yellow. (B) AF3-Multimer predicted structure of FOXK2 (pink) bound to WDR5 (blue). FOXK2 region predicted to interact with WDR5 is shown in yellow. (C) Predicted structure of WDR5, highlighting residues known to interact with the transcription factors (MYC, yellow), histones (K4me2, green) as well as predicted FOXK2 interface (red).

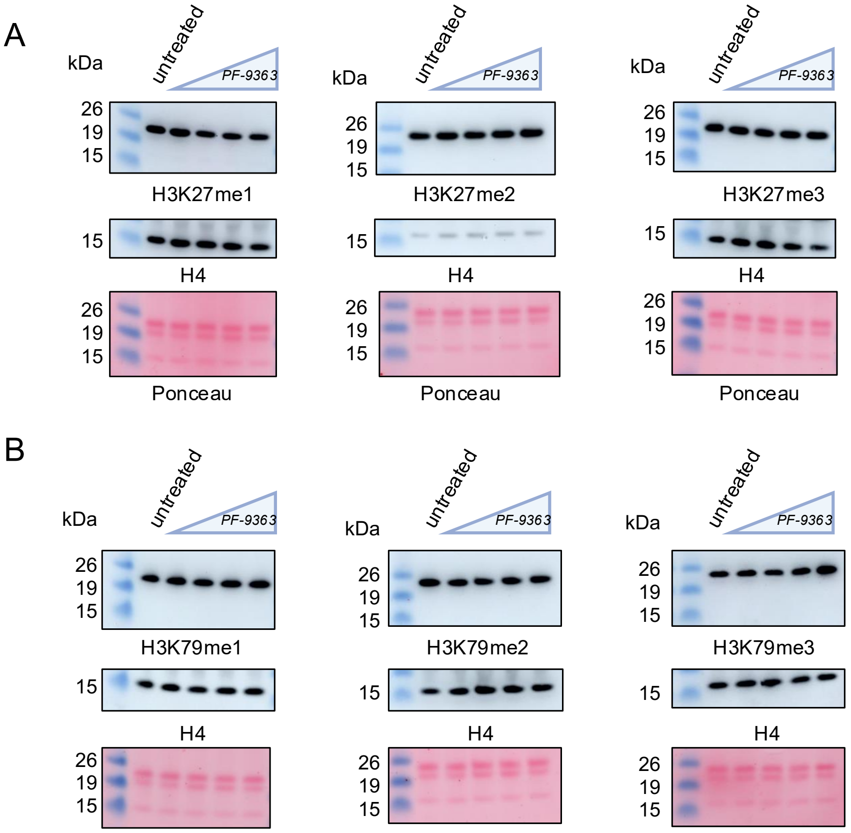

Figure S5. Analyzing the effect of PF-9363 on histone methylation. (A) Cellular response of H3K27 methylation to treatment with PF-9363. MCF-7 cells were treated with escalating dosages (0.1, 1, 10, 30 μM.) PF-9363 for 24 h. Data is representative of *n*=2 biological replicates. (B) Cellular response of H3K79 methylation to treatment with PF-9363. MCF-7 cells were treated with escalating dosages (0.1, 1, 10, 30 μM.) PF-9363 for 24 h. Data is representative of *n*=2 biological replicates.

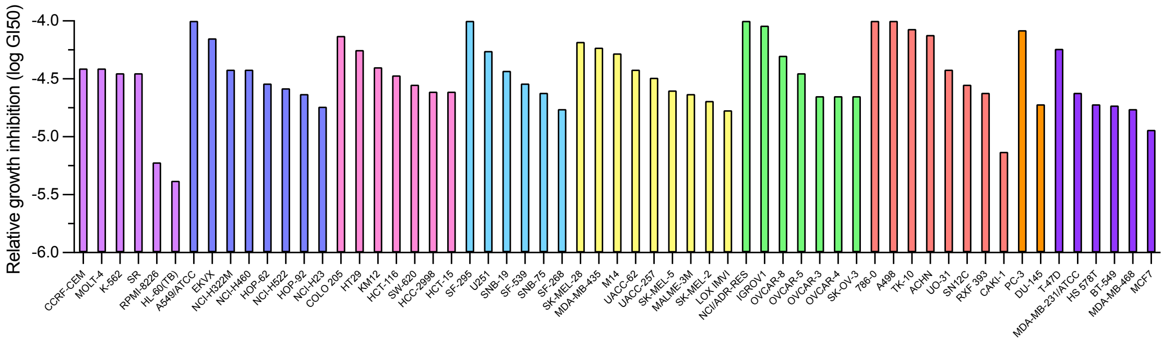

Figure S6. Half-maximal growth inhibition (GI_{50}) values for NCI-60 cell line panel treated with PF-9363 (48 h). Lower values correspond to more potent inhibition. Cell lines are colored by lineage as follows: light purple, leukemia; dark blue, non-small cell lung; pink, colon; light blue, CNS; yellow, melanoma; green, ovarian; red, renal; orange, prostate; dark purple, breast.

Uncropped scans of blots in supplementary figures

Figure S1B

Mag-capped beads: Elution anti-KAT2A

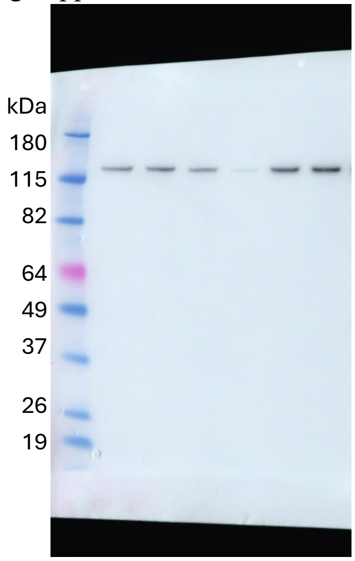

Flow through anti-KAT2A

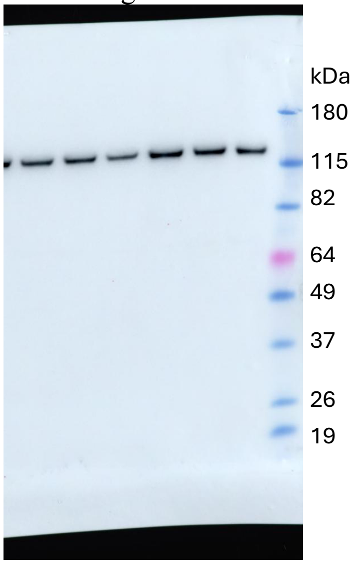

Input Ponceau

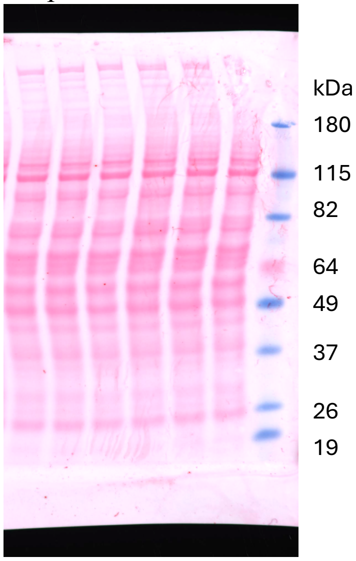

H3K14 CoA beads: Elution anti-KAT2A

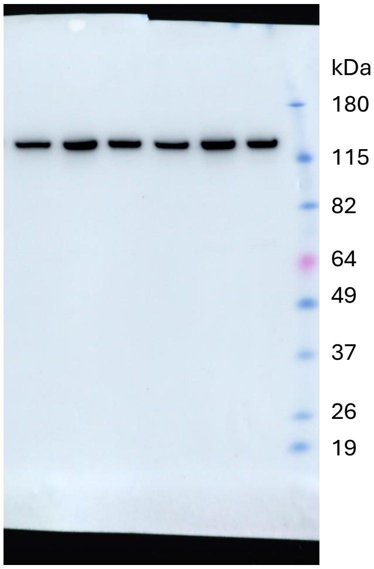

Flow through anti-KAT2A

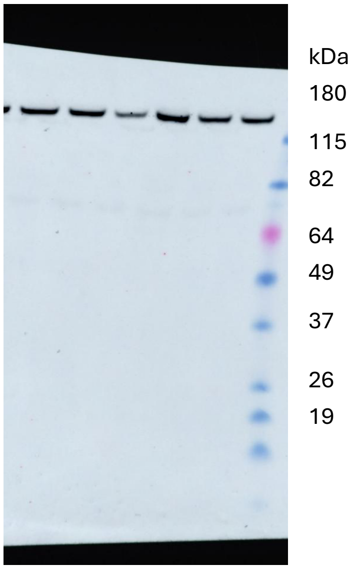

Input Ponceau

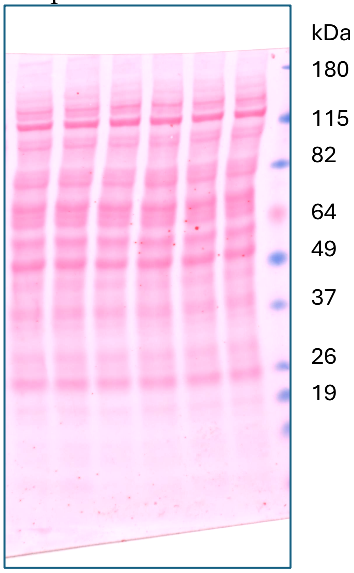

Figure S5

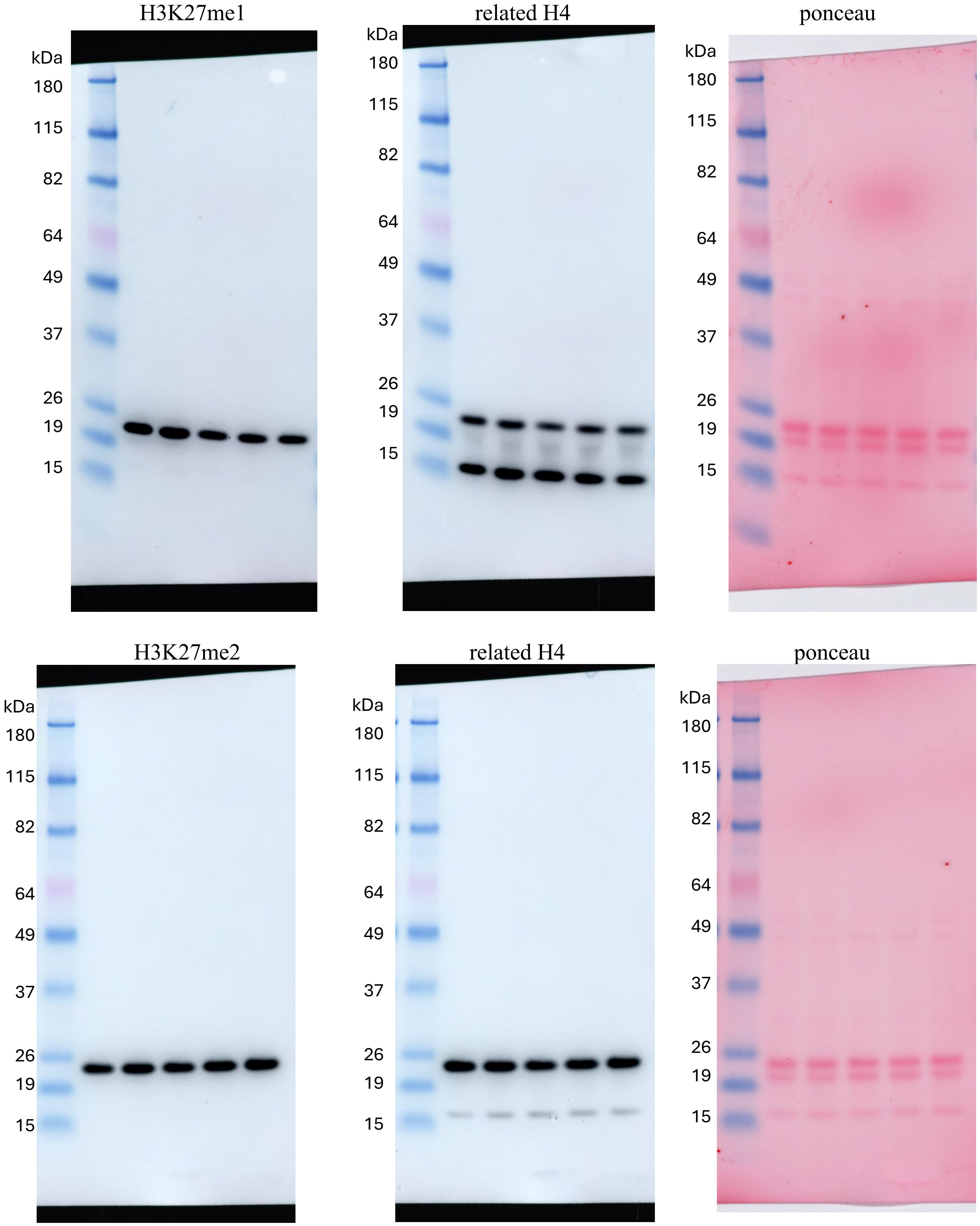
H3K27me1
kDa
180
115
82
64
49
37
26
19
15
related H4
ponceau
H3K27me2

H3K27me3

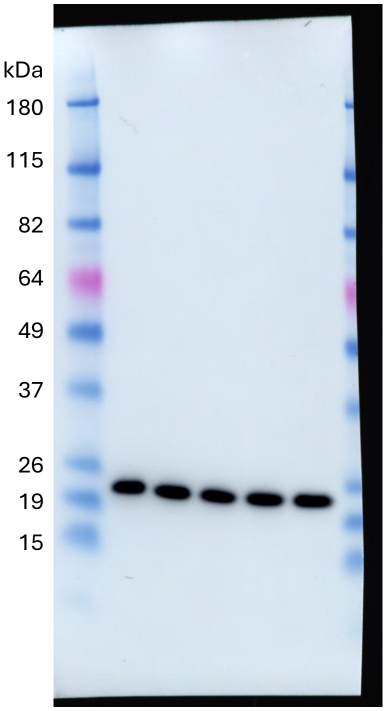

related H4

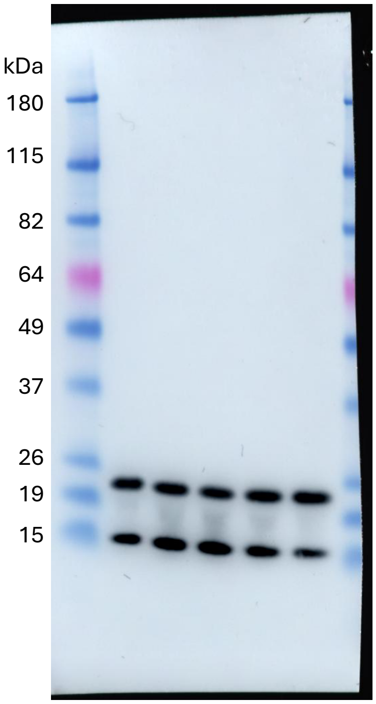

ponceau

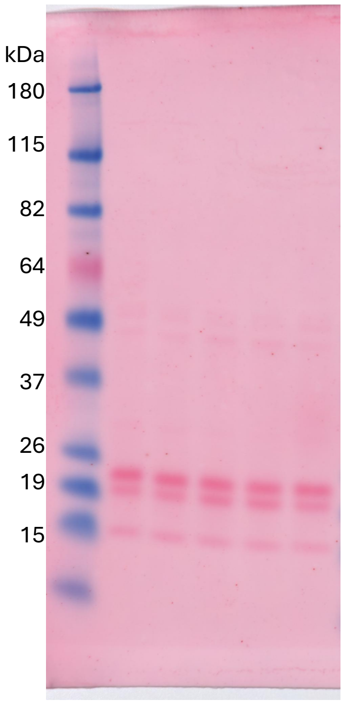

H3K79me1

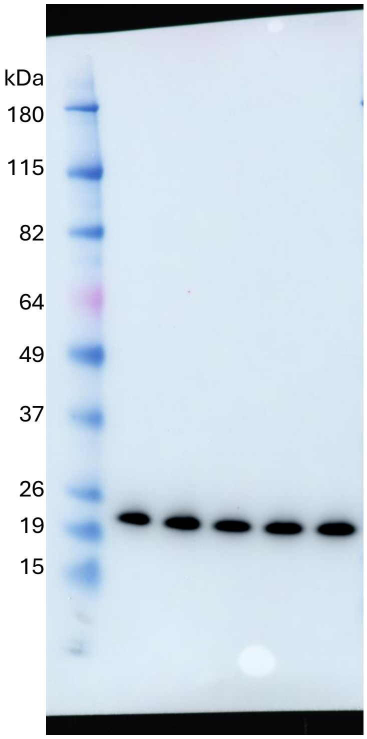

related H4

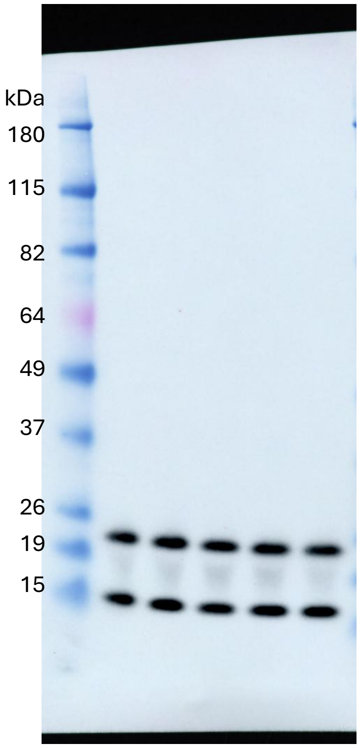

ponceau

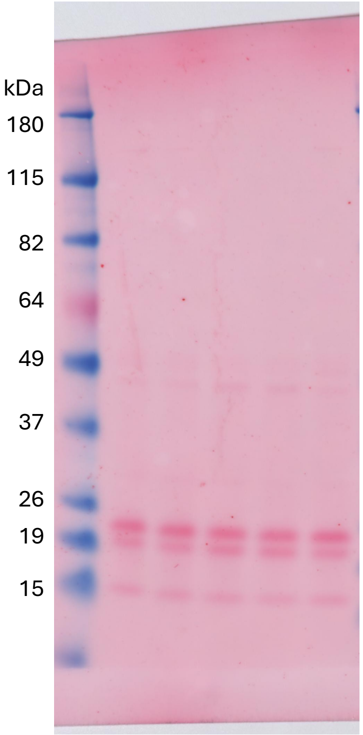

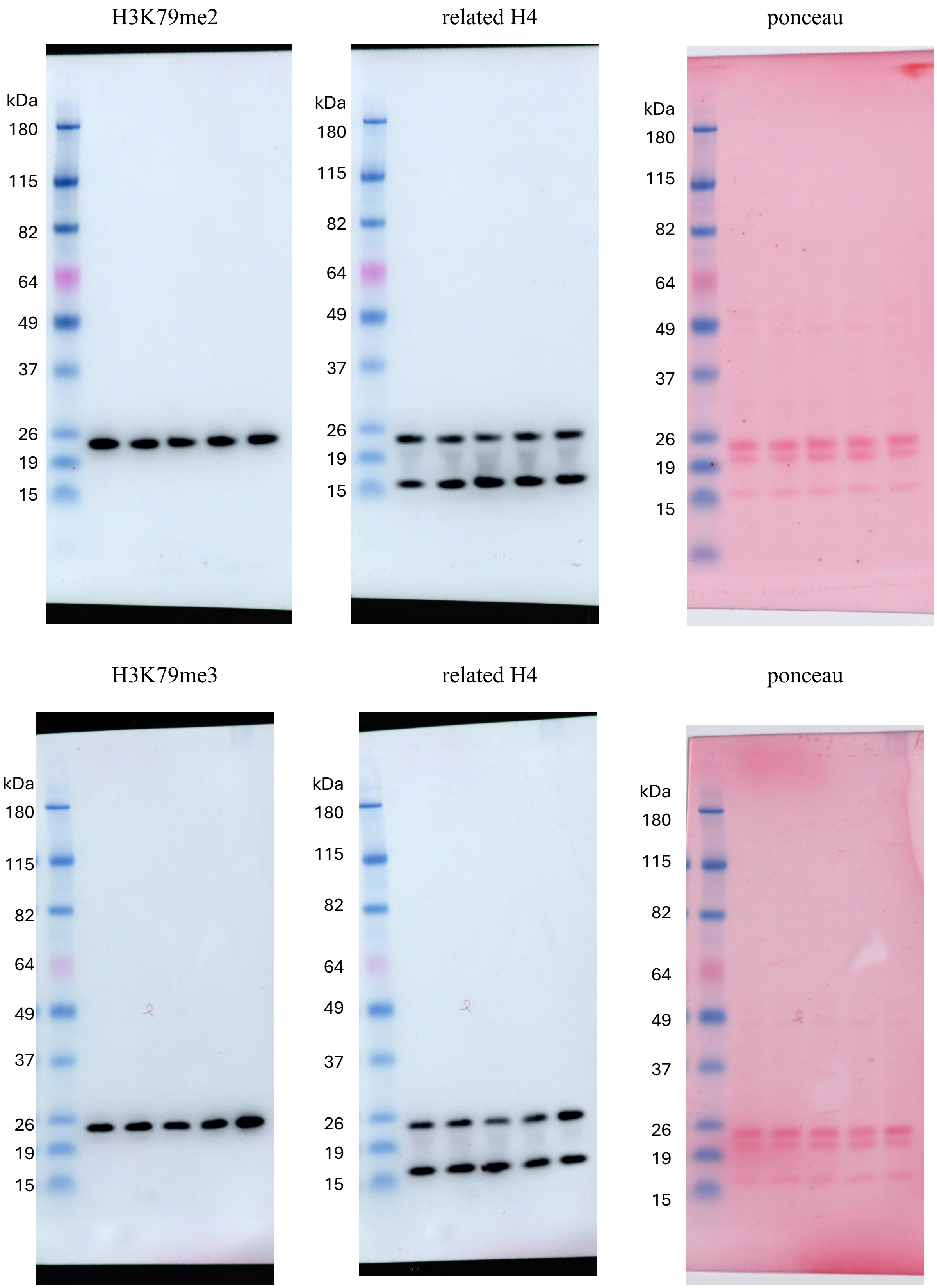
H3K79me2
related H4
ponceau
kDa
180
115
82
64
49
37
26
19
15
H3K79me3
related H4
ponceau
