# Supplementary material for: Hierarchical small molecule inhibition of MYST acetyltransferases
Source: Nat Commun. 2026 May 13;17:4329. doi: 10.1038/s41467-026-70574-1 (PMC13172425; doi:10.1038/s41467-026-70574-1)
Supplement: Supplementary file 2 — Description of Additional Supplementary Files [file 41467_2026_70574_MOESM2_ESM.pdf]

Description of Additional Supplementary Files

File name: Supplementary Data 1
Description: Reported MYST inhibitor selectivities. The assessment of different MYST inhibitors' selectivity for MYST enzymes/HAT domains from literatures.

File name: Supplementary Data 2
Description: MaxQuant analysis of protein abundance in Hela nuclear extracts by intensity-based absolute quantitation (iBAQ). Three replicates were performed.

File name: Supplementary Data 3
Description: Competitive profiling of different MYST inhibitors engaged proteome in KAT6A spiked-in Hela nuclear extracts. 5 μg of KAT6A (N-term Flag-tagged full-length hsKAT6A from Pfizer) was spiked into 1 mg of clarified Hela nuclear extracts (1 mg/mL) for per sample.1 μM of PF-9363 or Prifetrastat (PF-8144) was used to do competition. Three replicates for each treatment. The peptides and proteins were filtered at 1% false discovery rate. The p-values were calculated using a two-sided t-test with no adjustments for multiple comparisons.

File name: Supplementary Data 4
Description: Competitive profiling of PF-9363 engaged proteome in Hela nuclear extracts. 1 mL of 1 mg/mL nuclear extract was pre-incubated with a modified inhibitor concentration series (0, 0.1, 1, and 10 μM) of PF-9363 before KAT capture assay. Three biological replicates were performed for each treatment condition. The peptides and proteins were filtered at 1% false discovery rate. The p-values were calculated using a two-sided t-test with no adjustments for multiple comparisons.

File name: Supplementary Data 5
Description: Competitive profiling of WM-3835 engaged proteome in Hela nuclear extracts. 1 mL of 1 mg/mL nuclear extract was treated with a modified inhibitor concentration series (0, 0.1, 1, and 10 μM) of WM-3835 before KAT capture assay. Three biological replicates were performed for each treatment condition. The peptides and proteins were filtered at 1% false discovery rate. The p-values were calculated using a two-sided t-test with no adjustments for multiple comparisons.

File name: Supplementary Data 6
Description: t-distributed Stochastic Neighbor Embedding (t-SNE) clustering of proteins based on PF-9363 chemoproteomic competition profile (PF-9363 = 0, 0.1, 1, 10 μM). Unsupervised k-means clustering was subsequently applied with the number of clusters set to five.

File name: Supplementary Data 7
Description: AlphaFold/AlphaPulldown Analaysis of FOXK2 and protien binders reveal highest binders being OGT and WDR5.

File name: Supplementary Data 8
Description: Histone modification profiling upon treatment with escalating doses of PF-9363 (0, 0.1, 1, 10, 30 μM with MCF-7 cells) by bottom-up LC-MS/MS proteomics. Three biological replicates were performed for each condition.

File name: Supplementary Data 9
Description: DNA sequences used in plasmid construction for ectopic expression in the study of FOXK2 interactors. All plasmid constructs were ordered from TWIST, and pTwist CMV Puro backbone was used for all the plasmids. They all validated through Sanger and whole-plasmid sequencing.
