# Supplementary material for: Hierarchical small molecule inhibition of MYST acetyltransferases
Source: Nat Commun. 2026 May 13;17:4329. doi: 10.1038/s41467-026-70574-1 (PMC13172425; doi:10.1038/s41467-026-70574-1)
Supplement: Supplementary file 12 — Reporting Summary [file 41467_2026_70574_MOESM12_ESM.pdf]

nature portfolio

Corresponding author(s): Jordan L. Meier

Last updated by author(s): Feb 16, 2026

Reporting Summary

Nature Portfolio wishes to improve the reproducibility of the work that we publish. This form provides structure for consistency and transparency in reporting. For further information on Nature Portfolio policies, see our Editorial Policies and the Editorial Policy Checklist.

Statistics

For all statistical analyses, confirm that the following items are present in the figure legend, table legend, main text, or Methods section.

n/a	Confirmed	
☐	☒	The exact sample size (*n*) for each experimental group/condition, given as a discrete number and unit of measurement
☐	☒	A statement on whether measurements were taken from distinct samples or whether the same sample was measured repeatedly
☐	☒	The statistical test(s) used AND whether they are one- or two-sided<br>*Only common tests should be described solely by name; describe more complex techniques in the Methods section.*
☐	☒	A description of all covariates tested
☐	☒	A description of any assumptions or corrections, such as tests of normality and adjustment for multiple comparisons
☐	☒	A full description of the statistical parameters including central tendency (e.g. means) or other basic estimates (e.g. regression coefficient) AND variation (e.g. standard deviation) or associated estimates of uncertainty (e.g. confidence intervals)
☐	☒	For null hypothesis testing, the test statistic (e.g. F, t, r) with confidence intervals, effect sizes, degrees of freedom and P value noted<br>*Give P values as exact values whenever suitable.*
☒	☐	For Bayesian analysis, information on the choice of priors and Markov chain Monte Carlo settings
☐	☒	For hierarchical and complex designs, identification of the appropriate level for tests and full reporting of outcomes
☒	☐	Estimates of effect sizes (e.g. Cohen's *d*, Pearson's *r*), indicating how they were calculated

Our web collection on statistics for biologists contains articles on many of the points above.

Software and code

Policy information about availability of computer code

Data collection

All western blot Images were captured using an Amersham ImageQuant 800 imaging system (Cytiva, 29399482). TMT-based LC-MS/MS data and Label-free LC-MS/MS data were collected by a Dionex U3000 RSLC in front of a Orbitrap Eclipse (Thermo Scientific) equipped with an EasySpray ion source. The data of histone modifications was collected using a Vanquish Neo UHPLC coupled to an Orbitrap Exploris 240 mass spectrometer (Thermo Scientific). Luminescence signals were recorded on a BioTek Synergy 2 plate reader for cytotoxicity assay. Regarding in silico interaction screening, Binding structures between FOXK2 (Uniprot: Q01167) and candidate interactors identified from our chemoproteomic data were predicted using AlphaPulldown4 v0.30.7, with multiple sequence alignments generated via ColabFold Search v1.5.5 on the NIH HPC Biowulf Cluster. OGT (Uniprot: O15294) and WDR5 (Uniprot: P61964), were further analyzed to identify interface residue-residue contacts with FOXK2 using both AlphaFold2 and AlphaFold3. The code used to predict structure/interface contacts and assess FOXK2 interactors, and the results of structure prediction and interface contacts prediction in this study have been deposited to Zenodo (https://zenodo.org/records/15238967).

Data analysis

Raw MS files of TMT-based LC-MS/MS data were searched with Proteome Discoverer 2.4 using the Sequest node. Data was searched against the Uniprot Human database from August 2023. The raw files of Label-free LC-MS/MS data were searched against the human proteome database (UP000005640) from Uniprot (Accessed in November 2023) using Andromeda embedded in MaxQuant (version 2.4.13.0). Histone modification data were analyzed using EpiProfile 2.0. Half-maximal inhibition values (IC_{50}) were determined from nonlinear regression analysis of dose-response curves using GraphPad Prism 9 in Cytotoxicity assays. To prioritize potential interactions between FOXK2 and MYST components, we developed a composite scoring system integrating multiple AlphaFold metrics (mpDockQ/pDockQ, LIS, and LIA), building upon established approaches for protein-protein interaction analysis. Each interaction was evaluated against minimum acceptable thresholds (1610 for LIA, 0.073 for LIS, and 0.175 for mpDockQ/pDockQ) and assigned a weighted constant (k): 1.0 for passing all three thresholds, 0.75 for two, 0.5 for one, and zero for none. Metrics were then min-max normalized, summed, and multiplied by the weighted constant to generate final composite scores for ranking candidate interactions. For AlphaFold2 analysis we calculated Euclidean distances between alpha

carbon coordinates of residues using Sci-Py's Distance Matrix Module, filtering for interactions under 8Å. Highly interacting residues were defined as those with distances less than 6Å and PAE scores less than 25.7. For AlphaFold3 analysis we extracted the contact_probs metric from the summary JSON file, with highly interacting areas identified by contact probability values greater than 0.1.Predictions from both methods were compared to identify overlapping interface residues. The code used to predict structure/interface contacts and assess FOXK2 interactors in this study have been deposited to Zenodo (https://zenodo.org/records/15238967).

For manuscripts utilizing custom algorithms or software that are central to the research but not yet described in published literature, software must be made available to editors and reviewers. We strongly encourage code deposition in a community repository (e.g. GitHub). See the Nature Portfolio guidelines for submitting code & software for further information.

Data

Policy information about availability of data

All manuscripts must include a data availability statement. This statement should provide the following information, where applicable:

- Accession codes, unique identifiers, or web links for publicly available datasets
- A description of any restrictions on data availability
- For clinical datasets or third party data, please ensure that the statement adheres to our policy

The data supporting the findings of this study are available within the article and its Supplementary Figures. Structure prediction and interface contacts prediction data generated in this study have been deposited to Zenodo (https://zenodo.org/records/15238967). Raw mass spectrometry proteomics files and database search results have been deposited at the ProteomeXchange Consortium (http://proteomecentral.proteomexchange.org) with data set identifier "PXD074488". The accession code of crystal structure of PF-9363 bound to KAT6A catalytic domain is: 8DD5 (https://www.rcsb.org/structure/8DD5). Other data generated in this study are provided in the supplementary Information/supplementary tables /Sorce Data file.

Research involving human participants, their data, or biological material

Policy information about studies with human participants or human data. See also policy information about sex, gender (identity/presentation), and sexual orientation and race, ethnicity and racism.

Reporting on sex and gender	n/a
Reporting on race, ethnicity, or other socially relevant groupings	n/a
Population characteristics	n/a
Recruitment	n/a
Ethics oversight	n/a

Note that full information on the approval of the study protocol must also be provided in the manuscript.

Field-specific reporting

Please select the one below that is the best fit for your research. If you are not sure, read the appropriate sections before making your selection.

☒ Life sciences ☐ Behavioural & social sciences ☐ Ecological, evolutionary & environmental sciences

For a reference copy of the document with all sections, see nature.com/documents/nr-reporting-summary-flat.pdf

Life sciences study design

All studies must disclose on these points even when the disclosure is negative.

Sample size	Widely used sample sizes of 3 were employed for chemoproteomic studies. Sample sizes for the bioinformatic analyses performed is reported under the relevant section of online methods. Sample sizes of 2-4 was kinetic for immunoblotting and cell-based experiments as widely adapted in the field of chemical biology.
Data exclusions	No data was excluded.
Replication	All chemoproteomic analysis were performed in three independent experiments with one LC-MS/MS run each. All gel-based and immunoblotting experiments were performed as two or three independent experiments with similar results and representative images are shown in figures.
Randomization	Randomization was not performed because in each chemoproteomic experiment, only two groups of samples, under the indicated conditions were compared.
Blinding	We used isotopic labeling of the samples for quantitative chemoproteomic studies to compare MYST inhibitor engagement in parallel. Besides, we set capped beads group which couldn't enrich acetyltransferases to remove non-specific protein binding. Thus, it was not

necessary to design a blinding group allocation.

Reporting for specific materials, systems and methods

We require information from authors about some types of materials, experimental systems and methods used in many studies. Here, indicate whether each material, system or method listed is relevant to your study. If you are not sure if a list item applies to your research, read the appropriate section before selecting a response.

Materials & experimental systems

n/a	Involved in the study
☐	☒ Antibodies
☐	☒ Eukaryotic cell lines
☒	☐ Palaeontology and archaeology
☒	☐ Animals and other organisms
☒	☐ Clinical data
☒	☐ Dual use research of concern
☒	☐ Plants

Methods

n/a	Involved in the study
☒	☐ ChIP-seq
☒	☐ Flow cytometry
☒	☐ MRI-based neuroimaging

Antibodies

Antibodies used	The following primary antibodies (supplier name, catalog number, dilution times, manufacturer websites) were used: anti-KAT7 (Abcam, AB70183, 1:1000, https://www.abcam.com/en-us/products/primary-antibodies/kat7-hbo1-myst2-antibody-ab70183), anti-KAT8 (Cell Signaling, 46862S, 1:1000, https://awsprod-www.cellsignal.com/products/primary-antibodies/myst1-d5t3r-rabbit-monoclonal-antibody/46862), anti-Naa50 (Proteintech, 16120-1-AP, 1:1000, https://www.ptglab.com/products/NAT13-Antibody-16120-1-AP.htm), anti-Lamin A/C (Bethyl Laboratories, A303-431A, 1:2000, https://www.fortislife.com/products/primary-antibodies/rabbit-anti-lamin-a-c-antibody/BETHYL-A303-431), anti-H3K23ac (Millipore, 07-355, 1:10000, https://www.sigmaaldrich.com/US/en/product/mm/07355), anti-H3K14ac (Millipore, 07-353, 1:1000, https://www.sigmaaldrich.com/US/en/product/mm/07353), anti-H4K16ac (Millipore, 07-329, 1:1000, https://www.sigmaaldrich.com/US/en/product/mm/07329), acetyl-histone H2A.Z (Lys4/Lys7) (Cell Signaling, 75336, 1:1000, https://awsprod-www.cellsignal.com/products/primary-antibodies/acetyl-histone-h2a-z-lys4-lys7-d3v1i-rabbit-monoclonal-antibody/75336), anti-H3K18ac (Millipore, 07-354, 1:1000, https://www.sigmaaldrich.com/US/en/product/mm/07354), anti-H4 (Cell Signaling, 2935S, 1:1000, https://awsprod-www.cellsignal.com/products/primary-antibodies/histone-h4-l64c1-mouse-monoclonal-antibody/2935), anti-H3K27me (Cell Signaling, 84932, 1:1000, https://awsprod-www.cellsignal.com/products/primary-antibodies/mono-methyl-histone-h3-lys27-d3r8n-rabbit-monoclonal-antibody/84932), anti-H3K27me2 (Cell Signaling, 9728, 1:1000, https://awsprod-www.cellsignal.com/products/primary-antibodies/di-methyl-histone-h3-lys27-d18c8-rabbit-monoclonal-antibody/9728), anti-H3K27me3 (Cell Signaling, 9733, 1:1000, https://awsprod-www.cellsignal.com/products/primary-antibodies/tri-methyl-histone-h3-lys27-c36b11-rabbit-monoclonal-antibody/9733), anti-H3K79me (Cell Signaling, 12522, 1:1000, https://awsprod-www.cellsignal.com/products/primary-antibodies/mono-methyl-histone-h3-lys79-d5x1s-rabbit-monoclonal-antibody/12522), anti-H3K79me2 (Cell Signaling, 5427, 1:1000, https://awsprod-www.cellsignal.com/products/primary-antibodies/di-methyl-histone-h3-lys79-d15e8-rabbit-monoclonal-antibody/5427), anti-H3K79me3 (Cell Signaling, 74073, 1:1000, https://awsprod-www.cellsignal.com/products/primary-antibodies/tri-methyl-histone-h3-lys79-e8b3m-rabbit-monoclonal-antibody/74073), anti-Flag tag (Cell Signaling, 14793S, 1:1000, https://awsprod-www.cellsignal.com/products/primary-antibodies/dykddddk-tag-d6w5b-rabbit-monoclonal-antibody-binds-to-same-epitope-as-sigma-aldrich-anti-flag-m2-antibody/14793), anti-Myc tag (Cell Signaling, 2278S, 1:1000, https://awsprod-www.cellsignal.com/products/primary-antibodies/myc-tag-71d10-rabbit-monoclonal-antibody/2278), anti-rabbit IgG HRP-linked antibody (Cell Signaling Technology, 7074S, 1:1000) and anti-mouse IgG HRP-linked antibody (Cell Signaling Technology, 7076S, 1:1000).
Validation	All the commercial antibodies were validated by the manufacturers for species and application using recombinant proteins and/or expressing cell types as positive control; these validation studies are reported for each antibody on the product website.

Eukaryotic cell lines

Policy information about cell lines and Sex and Gender in Research

Cell line source(s)	MCF-7 , BT-549 and HEK-293T cells were obtained from the NCI tumor cell repository. Their catalog numbers are as follows: MCF-7 (ATCC # HTB-22), HEK-293T (ATCC # CRL-3216), and BT-549 (ATC # HTB-122).
Authentication	All cell lines were authenticated by ATCC by checking cellular morphology, karyotyping and short tandem repeat profiling.
Mycoplasma contamination	All cell lines used were tested and verified as free of mycoplasma contamination.
Commonly misidentified lines (See ICLAC register)	None is misidentified cell line by searching ICLAC register version 13 (released 26 April 2024)

Plants

Seed stocks	n/a
Novel plant genotypes	n/a
Authentication	n/a
